# Rising public interest in stem cell therapy for erectile dysfunction: an analysis of public perception and a review of the literature

**DOI:** 10.1177/17562872251322651

**Published:** 2025-02-24

**Authors:** Muhammed A. M. Hammad, Jake Miller, Mark I. Sultan, Elia Abou Chawareb, Hana S. Nakamura, Juan R. Martinez, Supanut Lumbiganon, Lawrence Jenkins, David W. Barham, Dhiresh Bandaru, Jessica M. Yih, Faysal A. Yafi

**Affiliations:** Department of Urology, University of California, 20 palatine, Apt 216 in Villa Sienna, Irvine, CA 92612, USA; Department of Urology, University of California, Irvine, Irvine, CA, USA; Department of Urology, University of California, Irvine, Irvine, CA, USA; Department of Urology, University of California, Irvine, Irvine, CA, USA; Department of Urology, University of California, Irvine, Irvine, CA, USA; Department of Urology, University of California, Irvine, Irvine, CA, USA; Department of Urology, University of California, Irvine, Irvine, CA, USA; Department of Surgery, Faculty of Medicine, Khon Kaen University, Khon Kaen, Thailand; Department of Urology, University of California, Irvine, Irvine, CA, USA; Department of Urology, University of California, Irvine, Irvine, CA, USA; School of Medicine, University of California, Riverside, Riverside, CA, USA; Department of Urology, University of California, Irvine, Irvine, CA, USA; Department of Urology, University of California, Irvine, Irvine, CA, USA

**Keywords:** alternative treatments, erectile dysfunction, epidemiology, evidence-based medicine, Google search trends, platelet-rich plasma, public interest, regenerative medicine, shockwave therapy, stem cell therapy

## Abstract

**Purpose:**

The use of alternative treatment modalities for erectile dysfunction (ED) beyond phosphodiesterase inhibitors continues to grow within the practice of Urology. Utilizing U.S. Google trends as a novel epidemiological tool for geographically associating patient search intent, our study aims to capture trends relating to interest in stem cell therapy (SCT) as a potential treatment for ED.

**Methods:**

An online search was conducted to identify centers in the United States offering stem cell therapy (SCT) for erectile dysfunction (ED), using specific keywords such as “ED treatment,” “stem cells for ED,” and “sexual health stem cell.” The geographic distribution of these centers was mapped, and their publicly available information was evaluated based on strict inclusion criteria, including direct claims of SCT efficacy for ED and oversight by a licensed urologist. The public interest in SCT treatment was quantified using Google Trends data from July 2018 to July 2023, utilizing search terms related to SCT and comparing them to terms associated with alternative regenerative therapies like platelet-rich plasma and shockwave treatments, to extract the direction and magnitude of national interest over the preceding 5 years. The PubMed, Cochrane Library, and EMBASE databases were then searched from inception to May 2024 regarding evidence for the use of SCT to treat ED.

**Results:**

Despite insufficient evidence, public search interest demonstrates an upward trajectory of this treatment when compared to alternative regenerative therapies for ED. This increased interest in SCT as a potential treatment option for ED may be linked to the marketing efforts of commercial entities. Throughout the qualitative analysis of advertisement sources, only two websites (Stem Cells Transplant Institute, and Ambrose Cell Therapy) summarized the collective results of a directed clinical trial investigating the utility of SCT in ED patients.

**Conclusion:**

Our study demonstrates the public prevalence of patients seeking SCT as a treatment modality for ED is increasing. In addition, varied sources nationwide promote SCT despite limited scientific evidence and consensus. This disparity calls for additional prospective research on the viability, efficacy, and long-term safety of SCT in the context of ED.

## Plain language summary

Rising interest in stem cell therapy for erectile dysfunction despite limited evidence

Erectile dysfunction (ED) is a common issue, and people are increasingly searching for new treatments beyond traditional medications. Our study looked at how many people in the U.S. are interested in stem cell therapy (SCT) as a potential treatment for ED, using Google search data to measure this interest. We searched medical databases for studies on SCT for ED and found that there isn’t enough scientific evidence to recommend it as a reliable treatment. However, our analysis of Google search trends over the past five years shows a growing public interest in SCT for ED, compared to other new treatments like shockwave therapy and plasma-rich platelet (PRP) therapy. This increasing interest in SCT might be due to aggressive marketing by clinics offering the treatment. These clinics often highlight the benefits of SCT, such as the simplicity of the procedure and success stories from patients, to make it seem more credible. However, they rarely mention that there is a lack of solid scientific proof supporting these claims. Our findings suggest that while many people are looking into SCT for ED, there is a significant gap between public interest and the scientific evidence available. This highlights the need for more research to determine if SCT is a safe and effective treatment for ED in the long term.

## Introduction

Erectile dysfunction (ED) is a multifaceted condition affecting over 100 million men worldwide. Defined as the inability to achieve or maintain a satisfactory penile erection for sexual intercourse, the incidence of ED rises in direct correlation with patient age and serves as an indicator of endothelial dysfunction.^1^ While ED is not a life-threatening condition, prior studies have demonstrated the negative impact of ED on factors including sexual intimacy, quality of life, productivity, psychological well-being, relationship building, and even work performance and productivity.^2^ As such, investigations into new, innovative treatments for ED remain an ongoing area of interest.

Standard treatments for ED include vacuum constriction devices, oral medications, vasoactive drug injection therapy, and more invasively, implantation of a penile prosthesis.^3^ However, a promising alternative therapy gaining attention for its utility within the treatment of ED is stem cell therapy (SCT). SCT aims to harness the regenerative potential of nascent, undifferentiated cells to offer a therapeutic benefit in the setting of endothelial dysfunction which potentiates ED.^4^

SCT remains a heterogeneous term encompassing totipotent cells which are undifferentiated cells capable of developing extraembryonic tissue, pluripotent cells which can form tissue in any embryonic germ cell layer, and multipotent cells which are restricted to developing tissue within a single germ cell layer.^5^ The introduction of SCT as a potential form of treatment remains an emerging topic offering utility for a plethora of medical conditions. However, the potential gains and the overall efficacy of such therapy in the context of ED remain relatively unknown. There have been limited trials to demonstrate SCT for ED generates favorable short-term results. The explored cell lines include adipose tissue-derived stem cells,^6^ bone marrow-derived stem cells,^7^ and embryonic stem cells.^8^ However, larger studies with a more robust longitudinal follow-up are warranted to accurately represent treatment efficacy, procedural standardization, and adverse effect profile.^9^ This aligns with the official position of the Sexual Medicine Society of North America, which asserts that additional multicenter randomized trials must be conducted before regenerative therapies can be accepted as standard practice.^10^ Nevertheless, a growing number of clinics in the United States continue to provide these treatments to patients with a minimal grasp of the potential long-term consequences.^11^ We, therefore, aimed to quantify the direction of public interest in SCT in the context of regenerative therapy modalities for ED and the availability of this treatment in the United States.

## Methodology

**Figure 1.**
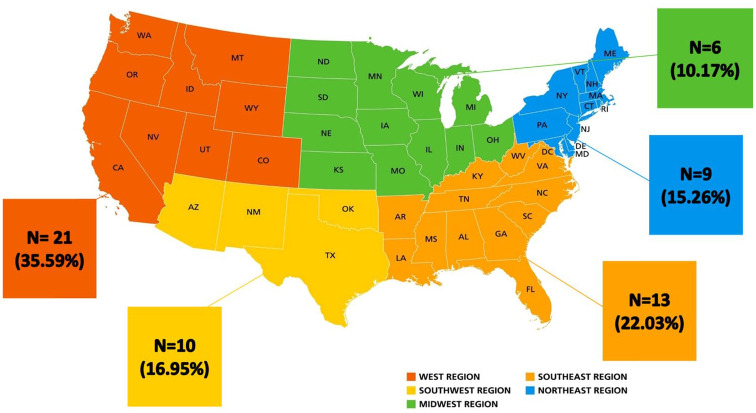
US map showing the location of major centers that provide stem cell therapy for erectile dysfunction.

An online search was conducted to identify centers in the United States offering SCT as a management option for ED. A map was then drawn to demonstrate the geographic distribution of all these centers across the United States (Figure 1). Through browsing the publicly available online information for these treatment centers, the quality of evidence offered regarding the efficacy of SCT in the management of ED was gathered. Criteria for inclusion were that a website directly states the use of SCT to alleviate active ED in addition to referencing success claims and that treatment was overseen by a licensed urologist, while exclusion criteria included sites not supervised by a licensed urologist. Keywords used to identify websites for our analysis were “ED treatment,” “stem cells for ED,” “innovative ED treatment,” “men’s health,” “men’s health stem cell,” “sexual health stem cell,” “ED solutions,” and “ED.” Only six centers met these strict criteria (Supplemental Table 1).

**Figure 2.**
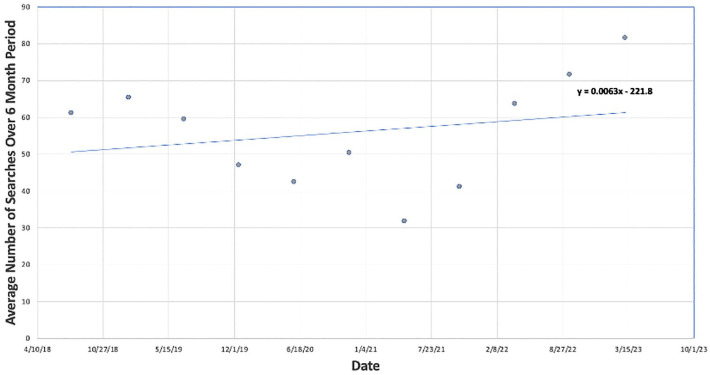
Google Trends average interest in the United States from 2018 to 2023: stem cell for erectile dysfunction.

**Figure 3.**
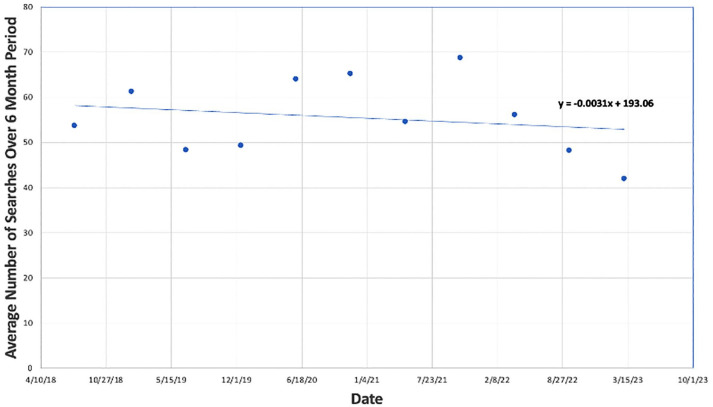
Google Trends average interest in the United States from 2018 to 2023: platelet-rich plasma for erectile dysfunction.

**Figure 4.**
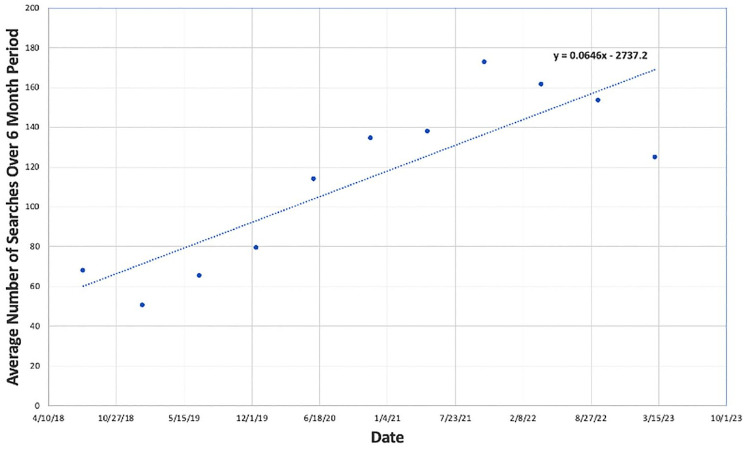
Google Trends average interest in the United States from 2018 to 2023: shockwave for erectile dysfunction.

Google Trends was then utilized to obtain data on search patterns for SCT as a treatment for ED between July 2018 and July 2023, with searches related to the weather used as a control to adjust for variations in internet usage.^12^ Search terms used to evaluate search interest in SCT included “ED treatment,” “men’s health,” “ED solutions,” “stem cells for ED,” “men’s health stem cell,” and “sexual health stem cell.” Running averages of the quantified search results every 24-week period (6 months) were then plotted against time (Figure 2). Search interest trends for the above terms were compared to terms relating to alternative regenerative therapies for ED, including “PRP for ED,” “ED shockwave therapy,” “shockwave for ED,” or “penile shockwave therapy” (Figures 3 and 4).

In addition, for a narrative review: PubMed, Cochrane Library, and EMBASE databases were searched from inception to May 2024 regarding evidence for the use of SCT to treat ED.

## Results

### Websites prescribing SCT for ED

Only six practicing clinics within the United States offered SCT as a treatment for ED while meeting the above-established criteria (Figure 1). Although a more significant number of clinics advertise SCT for ED, many of these establishments were excluded from our analysis because they were not run by licensed urologists. Instead, they were operated by practitioners from other specialties or by non-physician providers. At the clinics that met the criteria, the average out-of-pocket costs associated with SCT ranged between 5 and 10,000 dollars, with patients being offered on average a treatment course consisting of three sessions. The offered cell lines advertised to be most efficacious included umbilical-derived, adipose-derived, bone marrow-derived, or blood-derived stem cells, with most of the identified locations advertising umbilical-derived stem cells.

Throughout the qualitative analysis of advertisement sources, it was recognized that all six commercial website pages that met the described inclusion criteria evidenced the effectiveness of stem cell therapy by quoting observed patient testimonials. Only two websites (Stem Cells Transplant Institute, and Ambrose Cell Therapy) summarized the collective results of a directed clinical trial investigating the utility of SCT in ED patients.

### National interest for SCT to manage ED

Nationwide interest in the use of SCT to treat ED, as described by consumer Google Trends between July 2018 and July 2023, is represented in Figure 2. Data quantifying consumer interest from Google Trends was fractioned over searches related to the climate from each respective metropolitan area, denoted as Metros on Google Trends software. The Metros were then averaged across the nation to quantify interest. Over a 5-year-span, average search interest in search trends relating to the use of SCT for the treatment of ED increased. Notably, the data demonstrate a decrease in interest from late 2019 through 2021, aligning with the onset of the COVID-19 pandemic, associated lockdowns, and limited access to ambulatory healthcare. However, beginning in 2022, interest in SCT demonstrated a rebounding and consistently increasing trend. In fact, since 2021, the Google Trends for SCT has been the only regenerative medicine solution for ED which has demonstrated increasing public interest searches in comparison to those for platelet-rich plasma (PRP) and shockwave therapy.

### National interest in PRP and shockwave therapy to manage ED

The Google Trends of the last 5 years for PRP treatment-related searches regarding ED are represented in Figure 3. PRP national interest demonstrated a decline beginning in 2019, though demonstrated a modest resurgence from 2020 through 2021. However, beginning in late 2021, interest in PRP treatments demonstrated a consistent downward trend. By creating a linear trend of the 5 years of collected data, public interest in PRP therapy demonstrates a downward trajectory.

Data extracted from Google Trends regarding the national interest in the use of shockwave therapy treatment of ED from July 2018 to July 2023 is represented in Figure 4. A consistent and gradual increase in search interest for terms relating to shockwave therapy as a treatment of ED was noted. However, starting in the latter half of 2021, interest in shockwave therapy begins to decrease in search trends.

## Discussion

Our study aimed to explore centers offering SCT management for ED as well as investigate public interest in SCT for ED across the United States by using Google Trends as an epidemiological tool. The results demonstrate an increasing trend for stem cell-related searches to manage ED, indicating significant public interest in this emerging treatment. The limited number of clinics that met our criteria—only six nationwide—highlights a potential mismatch between public interest and the availability of reputable providers. This suggests that many patients may be seeking treatment from providers who do not meet established standards, raising concerns about the quality and safety of care being received. This interest may be attributed to the marketing efforts of commercial entities promoting SCT which advertise its appealing benefits and procedural tolerability through patient testimonials beyond the current standard of care.

Our study provides valuable insights for the medical community by highlighting the growing disconnect between patient interest and clinical evidence for SCT in ED. By uncovering this gap, we emphasize the need for healthcare professionals to proactively address patient inquiries, ensuring that individuals receive accurate information and understand the current limitations of SCT as a treatment option.

The high costs associated with SCT, averaging between 5 and 10,000 dollars for a course of treatment, indicate a significant financial burden on patients. Despite this, the willingness of patients to invest in SCT suggests a strong desire for alternative treatments, possibly due to dissatisfaction with existing therapies or the hope for a more permanent solution. Since 2021, the Google Search Trends for SCT has been the only regenerative medicine solution for ED which has demonstrated increasing public interest in comparison to those for PRP and shockwave therapy. This shift may reflect changing perceptions of efficacy or novelty among these therapies, with SCT being viewed as the most promising option despite limited clinical evidence.

It is crucial to also consider how the COVID-19 pandemic may have influenced search behaviors by heightening the general interest in health awareness and looking up new modalities for sexual health enhancement. In addition, with lockdowns and a decrease in in-person medical consultations, there could have been a shift to online searches for shockwave therapy and SCT as people sought additional methods to address health issues privately and remotely. The pandemic could have also induced significant psychological and mental stress which could exacerbate or manifest as ED. The increasing rates of anxiety and depression in conjunction with the previous factors discussed may have impacted ED search queries during the pandemic.^2^ Furthermore, the reliance on patient testimonials as the primary evidence of efficacy on commercial websites—rather than peer-reviewed clinical studies—raises concerns about the potential for misinformation. This emphasizes the need for regulatory oversight and for clinicians to guide patients toward evidence-based treatments.

Our analysis indicates that patients may not receive adequate information from these websites to make informed decisions. The lack of peer-reviewed evidence and the emphasis on anecdotal testimonials may mislead patients regarding the efficacy and safety of SCT for ED. This underscores the responsibility of healthcare providers to educate patients about the current state of research and to caution them against unverified treatments.

To further examine the current state of SCT for the treatment of ED, we further conducted a narrative review of its use.

### Review of the literature

In recent years, stem cells derived from either the bone marrow or umbilical cord have garnered interest in clinical trials aimed at treating several disorders.^13^ Though the results remain inconclusive, a minor or transitory improvement has been reported and corroborated for the treatment of ED.^13^ Within the scope of managing ED, only 18 studies aimed to delineate the efficacy and practicality of SCT have been conducted. Matz et al. assessed the association between ED and the utility of a variety of different stem cell sources including those derived from adipose tissue, bone marrow, placental, and urinary sources.^9^ The results demonstrated stem cell acquisition was more favorable for cells of urinary and placental origin. These reported results were in addition to a review of numerous clinical trials reporting a benefit in the treatment of ED. However, it is important to note that the study addresses the uncertainty of these results and that future research ought to be conducted.^9^

In a systematic review of human trials using SCT for ED by Lokeshwar et al., 61 patients who participated in either a phase I or phase II clinical trial were followed for up to 62 months. The majority of the studies demonstrated superior erectile function gauged by improved penile vascular flow, International Index of Erectile Function scores, and Erectile Hardness Scale scores.^14^ Within the context of the reported studies, no serious adverse events were reported. However, limitations included a small cohort size warranting additional investigations to ensure safety, consistent efficacy, and treatment standardization.^14^ Another review by He and Schwarz reviewed 27 trials for SCT to treat ED. Their results demonstrate three trials were subsequently withdrawn and the other trials were either incomplete or not yet published. Therefore concluding insufficient data to safely make concrete correlations regarding the safety and efficacy of SCT in the treatment of ED.^15^

After a narrative review of the literature, it is shown that SCT may prove advantageous for treating patients with ED. However, limited prospective randomized trials exist to significantly corroborate a benefit.

Though limited evidence exists to demonstrate the utility of SCT in the context of ED, stem cells have been used to treat a plethora of other diseases such as spinal cord paralysis. Preliminary results have shown promise within a regenerative neural context to ameliorate impairment after trauma for neurological networks.^16^ However, treatment benefits may be implicated by factors such as the cell line used, dosing, and transplantation timing. Cell replacement therapy has also been conducted regarding alleviating prevailing symptoms within Parkinson’s disease and several trials have reported the introduction of SCT resulting in adequate symptom relief for a subset of participants with over a decade of follow-up data.^17^ In the context of liver disease, SCT provides additional benefits for treating liver failure. Both mesenchymal stem cell-derived hepatocytes and mesenchymal stem cells transplanted through intrasplenic, or intravenous routes differentiated into functional hepatocytes.^14^ However, intravenous administration appeared superior, thus demonstrating many variables that may impact the results of therapy and highlighting the importance of considering an optimized treatment technique and therefore warranting additional studies.^18^ Hence, SCT is understood to not only provide benefit to tissue function through paracrine signaling but also by direct revitalization and replacement of damaged cells.^19^ In the context of disease processes attributed to endothelial dysfunction, such as ED, SCT may then offer tremendous utility.

SCT implementation for ED has progressively become more prevalent with increased advertisement efforts by commercial entities. Through a qualitative assessment of commercial marketing via the examination of websites promoting SCT to treat ED, we found that businesses provided an extensive array of benefits attributed to SCT while emphasizing the minimally invasive nature of the procedure. Of note, treatment did not appear standardized between the advertisement sources. Therefore, a lack of consensus remains regarding the procedural approach. Patient testimonials were often used to add credibility to the treatments offered. However, relying solely on anecdotal patient testimonials may potentiate the possibility of inaccurately represented outcomes due to potential bias, misinformation, or selective reporting of positive results.

The increasing advertising of SCT without substantial clinical evidence raises ethical concerns. This trend is worrisome from the authors’ perspective, as it may lead to patient harm due to unproven treatments and erode trust in the medical profession. There is an urgent need for regulatory bodies to monitor and regulate such advertising to prevent the dissemination of misleading information.

These results further highlight the need for randomized prospective trials to determine the utility of SCT, as the current level of evidence is insufficient for both patients and the scientific community to make informed decisions and build an appropriate yet standardized procedural approach.

Additional clinical trials and robust prospective research remain indispensable to establish the safety and efficacy of SCT prior to physician recommendation. Moreover, potential ethical implications and challenges associated with regulating the marketing of unproven therapies must be considered. Regulatory bodies and healthcare professionals ought to collaborate to ensure patients receive accurate, evidence-based information regarding the potential benefits and risks of SCT for ED.

Our study indicates the importance of open communication and education for healthcare providers and patients. Providers should inform patients about the current evidence gaps and potential risks associated with SCT for ED. Patients and their partners should be encouraged to critically assess online information and consult with qualified healthcare professionals before pursuing such treatments.

Urologists should be aware of these trends and not necessarily be afraid but somewhat proactive. They should stay informed about the latest developments, participate in research, and educate their patients about the evidence-based options for ED treatment. By doing so, they can help mitigate the risks associated with unproven therapies and support patients in making informed healthcare decisions.

These findings underscore the need for randomized prospective trials to evaluate SCT’s utility, as current evidence is insufficient for informed decision-making by patients and the scientific community. Additional clinical trials are essential to establish SCT’s safety and efficacy before physician recommendation. Furthermore, ethical implications and challenges in regulating the marketing of unproven therapies must be addressed. Regulatory bodies and healthcare professionals should collaborate to provide accurate, evidence-based information about SCT for ED.

Limitations of our study include the use of Google Trends as the sole epidemiological tool, which may not capture the full scope of public interest in SCT for ED, as Google Trends only reflects online search behavior. In addition, our study did not explore the role of social media and other online platforms in shaping public opinion and interest in this treatment option. In addition, differences in protocols for SCT administration for SCT administration that were reported on the various websites do limit the ability to comment on potential standardization of care.

## Conclusion

Our study explores the status of SCT for ED in the United States by studying the association between the growing public interest in SCT for ED and the US sites offering this therapy, despite the limited available literature. Additional prospective trials are therefore needed to establish the safety and efficacy of this treatment modality. Healthcare professionals and regulatory bodies must aim to ensure patients are well-informed about the potential benefits and risks associated with SCT for ED. Future endeavors also ought to explore the factors driving potential regional differences in public interest as well as the role of social media and other online platforms in shaping public opinion for this treatment option.

In conclusion, the medical community should recognize the disconnect between patient interest and clinical evidence regarding SCT for ED. There is a clear need for enhanced patient education, regulatory oversight, and rigorous clinical research to ensure patient safety and uphold the integrity of medical practice.

## Supplemental Material

sj-docx-1-tau-10.1177_17562872251322651 – Supplemental material for Rising public interest in stem cell therapy for erectile dysfunction: an analysis of public perception and a review of the literature Supplemental material, sj-docx-1-tau-10.1177_17562872251322651 for Rising public interest in stem cell therapy for erectile dysfunction: an analysis of public perception and a review of the literature by Muhammed A. M. Hammad, Jake Miller, Mark I. Sultan, Elia Abou Chawareb, Hana S. Nakamura, Juan R. Martinez, Supanut Lumbiganon, Lawrence Jenkins, David W. Barham, Dhiresh Bandaru, Jessica M. Yih and Faysal A. Yafi in Therapeutic Advances in Urology

## Declarations

**Ethics approval and consent to participate:** Ethical approval was not required, as there are no human participants in this article and informed consent is not required.

**Consent for publication:** Not applicable.

**Availability of data and materials:** The data that support the findings of this study are available on request from the corresponding author.
