# Supplementary material for: Rising public interest in stem cell therapy for erectile dysfunction: an analysis of public perception and a review of the literature
Source: Ther Adv Urol. 2025 Feb 24;17:17562872251322651. doi: 10.1177/17562872251322651 (PMC11848873; doi:10.1177/17562872251322651)
Supplement: sj-docx-1-tau-10.1177_17562872251322651 – Supplemental material for Rising public interest in stem cell therapy for erectile dysfunction: an analysis of public perception and a review of the literature [file sj-docx-1-tau-10.1177_17562872251322651.docx]

Supplementary Table 1: Description of the Websites of Centers that Provide SCT for ED

| Innovations Medical | The website relies on testimonials formatted as videos providing a larger sense of credibility, for their target audience, for the effectiveness of their product. Information was also displayed through diagrams |
| --- | --- |
| Stem Cells Transplant Institute | Main source of evidence used to demonstrate the effectiveness of their product is through implementation of a research paper conducted by researchers from the Odense University Hospital. To create a greater sense of trust/advertisement for people obtaining their product is through 13 video testimonials from individuals who had used the product. |
| Maze Men’s Health | The website includes testimonials and expert videos. It provides Q&A section with some references. The center is led by a board-certified urologist. |
| Ambrose Cell Therapy | Describes the benefit their product has and provides a link to the sources. Regarding their form of advertisement, they rely on client testimonials in which everyone has an entire section of their story describing the overall benefits of their product. Also provide a link for peer reviewed paper, podcasts, articles, and patient brochures providing further explanations |
| Regenerative Medicine Center of Southern California | Describes their products FDA status as well as describes the benefits of their product. Regarding advertisement what is used are testimonials provided by past clients. Brochure and blog are also provided to provide further information |
| Regenerative and Sports Medicine | Site describes the overall benefits their products have. As such advertisement is achieved through the sites itself stem cell section in which the benefits are listed. |
